# Test–Retest Reliability of Cervical Strength Testing Protocols with Handheld Dynamometer in Prepubertal and Pubertal Untrained Boys

**DOI:** 10.3390/jfmk10020173

**Published:** 2025-05-14

**Authors:** Christos Batatolis, Konstantina Karatrantou, Theodora Vasilopoulou, Konstantina Chanou, Nikolaos Tsiakaras, Vassilis Gerodimos

**Affiliations:** Department of Physical Education and Sport Science, University of Thessaly, 42100 Trikala, Greece; batatoli@uth.gr (C.B.); kokaratr@uth.gr (K.K.); vasilopoulouphys@gmail.com (T.V.); konchanou@gmail.com (K.C.); ntsiakar@uth.gr (N.T.)

**Keywords:** reproducibility, isometric evaluation, neck, developmental years

## Abstract

**Background**: The cervical spine plays an important role in several daily activities of children and adolescents, and thus, its evaluation using reliable protocols is of crucial importance. This study examined the test–retest reliability of cervical strength protocols using indices of absolute (standard error of measurement—SEM and 95% limits of agreement—LOA) and relative reliability (intraclass correlation coefficient—ICC). **Methods**: Twenty prepubertal (9.10 ± 0.61 years old) and twenty pubertal boys (13.6 ± 0.6 years old) participated in two assessment sessions separated by 48 h. During both sessions, maximal isometric strength (forward flexion, extension, and right–left lateral flexion) was assessed using a handheld dynamometer, and then, the cervical flexion-to-extension ratio (cervical_F/E_ ratio) was calculated. **Results**: According to our data analysis, good absolute and relative reliability was denoted for prepubertal boys in all cervical movements (ICC = 0.81–0.90; SEM% = 5.82–8.62); conversely, pubertal boys denoted high relative and absolute reliability in all directions of movements (ICC = 0.90–0.96; SEM% = 3.8–5.5). The cervical_F/E_ ratio showed moderate reliability in prepubertal (ICC = 0.71; SEM% = 9.11) and pubertal boys (ICC = 0.78; SEM% = 7). **Conclusions**: In conclusion, the isometric strength of cervical muscles, using a handheld dynamometer, showed acceptable reliability in prepubertal and pubertal boys; however, prepubertal boys demonstrated slightly lower reliability. Additionally, the assessment of the cervical _F/E_ ratio should be interpreted with caution. However, it would be important to carry out future studies to strengthen the findings of the present study.

## 1. Introduction

Cervical pain is a common musculoskeletal problem in children and adolescents, with incidence ranging from 27 to 87% [1,2,3,4,5]. There is evidence that increased strain on the cervical spine may lead to cervical degeneration along with other developmental, medical, psychological, and social complications during the developmental years [1], while cervical spine injuries may lead to mortality (mortality rate: 4.2–4.5%) in children and adolescents [6,7]. Different factors that tend to increase the prevalence of cervical pain during the developmental years include sedentary activities (increased screen time, especially excessive mobile phone use) [4,5,8] and/or bad ergonomic conditions during daily life such as improper sitting position [2], as well as detrimental posture in cervical flexion during smartphones use, school furniture features (desk height and/or seat pan inclination) [2,9,10], and inappropriate schoolbag weight [3,10].

Unlike the factors mentioned above, which can cause detrimental effects (pain and injuries) on the cervical spine, other factors can act protectively (i.e., physical activity and exercise). In more detail, Myrtveit et al. [11] found that engaging in physical activity is related to a reduced risk of neck and shoulder pain in adolescents. In contrast, other studies have found no association between physical activity and neck–upper limb pain [12,13]. Furthermore, several previous studies have reported a decrease in neck pain after specific muscle-strengthening programs [14,15,16,17] in different populations, indicating the importance of muscle strength to the protection of the cervical spine. It should be mentioned that the evaluation of cervical strength using reliable testing protocols may be one of the most important steps for the successful design and implementation of safe and efficient muscle strength programs for the cervical spine.

In the scientific literature, there are several studies on adults that have examined the reliability of cervical strength measurement using fixed machines, isokinetic dynamometers, or fixed strain gauges reporting good–high reliability (ICC = 0.83–0.99) [18,19,20,21,22]; conversely, in the developmental years, the information is limited. More specifically, two studies that examined the reliability of the cervical strength in children and/or adolescents during forward flexion, extension, and/or rotation with fixed machines reported high reliability (ICC > 0.90) [23,24]. Moreover, one study [25] that investigated the reliability of a cervical strength test protocol by using a fixed loadcell reported good to high reliability (ICC = 0.86–0.92) in adolescent rugby athletes; conversely, a previous study [26], which examined the reliability of maximal isometric strength by using an isokinetic dynamometer in athletes with multiple concussions and healthy athletes of developmental ages, denoted good reliability (ICC = 0.89–0.90).

Over the last few years, handheld dynamometers have gained great popularity in the evaluation of strength due to their ease of use in different sports and clinical settings, convenient size, portability, accessibility, and overall low cost [27]. To date, many studies have examined the reliability of testing protocols with handheld dynamometers in various body parts (i.e., wrist, elbow, shoulder, knee, hip, ankle, or cervical spine) [28,29,30,31,32,33], reporting inconsistent results depending on the tested body part. Regarding the cervical spine, several researchers [32,33,34,35,36,37,38,39] have investigated the test–retest reliability of various testing protocols in healthy adults or in adults with neck pain, migraines, and whiplash injuries, demonstrating moderate to high reliability (ICC = 0.63–0.99, depending on the study and the protocol used). On the other hand, to the best of our knowledge, for the developmental years, the scientific knowledge regarding the reliability of cervical strength measurement using handheld dynamometers is very limited. Specifically, there is only one study that evaluated the reliability of cervical strength protocols (forward flexion and extension) by using a handheld dynamometer in healthy children and adolescents, as well as in children and adolescents with spinal muscular atrophy, that has reported high reliability (ICC = 0.98–0.99) [40].

It should also be mentioned that in the above study by Stepien et al. [40], a mixed sample of children and adolescents (5–16 years old) was used; no previous study has separately examined the reliability of cervical strength measurement using a handheld dynamometer on children and adolescents. The examination of reliability separately by age group may be of crucial importance since the reliability of measurement could be influenced by age [41,42]. Differences in mood, motivation, learning effect, the ability to focus on the task, and biomechanical factors may account for these age-related differences in reliability [41,42].

Taking all the above into consideration, the main objective of this study was to examine the test–retest reliability (using relative and absolute reliability indices) of maximal isometric cervical strength (a. extension, b. forward flexion, c. right–left lateral flexion, and d. cervical flexion-to-extension ratio) by using the Kinvent handheld dynamometer, separately, on prepubertal and pubertal untrained boys (comparing the reliability and the cervical strength values between the two age groups). Furthermore, we also examined the test–retest reliability to the total sample (mixed sample of prepubertal and pubertal untrained boys).

## 2. Materials and Methods

### 2.1. Participants

In total, 20 prepubertal (8.01–10.2 years old) and 20 pubertal (12.8–14.8 years old) untrained boys participated voluntarily in this study (Table 1). Participants were excluded from the study if they were systematically involved in sports or if they showed any pain or were injured in the cervical area in the last 6 months. Before the initiation of the study, the participants and their parents were informed about the testing procedures and signed a consent form. This study was conducted according to the Declaration of Helsinki and approved by the Ethics Committee of the University of Thessaly (approval number: 2083; date of approval: 8 February 2023).

### 2.2. Study Design

A week before the initiation of the study, the participants were informed about the evaluation procedures and were familiarized with the equipment and the testing protocols. During the familiarization session, the participants performed 3 submaximal trials in each direction of movement (forward flexion, extension, and right and left lateral flexion). Furthermore, on the same day, basic anthropometric characteristics were measured, and biological age was estimated. Following the familiarization session, two assessment sessions (test–retest) were carried out 48 h apart, and maximal isometric cervical strength during forward flexion, extension, and right and left lateral flexion was evaluated. Both assessment sessions were conducted at the same time of the day under the same conditions and by the same investigator. During both test and retest sessions, before the initiation of the main testing protocol, the participants performed a warm-up protocol. During the warm-up protocol, the participants were asked to sit with their backs straight, their hands on the thighs of their legs, and with their feet placed flat on the floor. From this position, they performed (a) a dynamic range of motion movements (forward flexion, extension, lateral flexion, and rotation; 1 set × 10 repetitions per direction); (b) static stretching exercises (forward flexion, extension, and lateral flexion; 1 set × 10 s per direction); and (c) a single self-resistance isometric hold (5 s) for each of those movement directions.

### 2.3. Testing Procedures

#### 2.3.1. Anthropometric Characteristics and Biological Age

Body mass and body height were assessed using a calibrated physician’s scale (Seca model 755, Seca, Hamburg, Germany) and a telescopic height rod (Seca model 220, Seca, Hamburg, Germany), as previously described by the American College of Sports Medicine (ACSM) [43]. In more detail, during body mass measurement, the participant, lightly clothed and without shoes, stood in the center of the weight scale with his body weight equally distributed on both feet, his hands free at his sides, looking straight ahead [43]. The body mass measurement was repeated twice and was performed with an accuracy of 0.5 kg. During body height measurement, the participant stood without shoes, with his weight equally distributed on both feet and his arms hanging freely at his sides [43]. The feet (joined), the head (standing), the shoulder–back, the buttocks, and the heels rested against the telescopic height rod. The body height measurement was repeated twice and was performed with an accuracy of 0.5 cm. Furthermore, biological age was self-estimated through Tanner’s sexual maturation stages (I, II, III, IV, and V) and was determined according to pubic hair development [44].

#### 2.3.2. Cervical Strength

**Figure 1:**
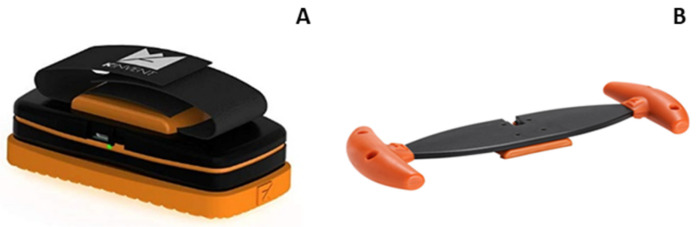
Kinvent K-force handheld dynamometer (**A**) and twin handles (**B**).

Maximal isometric cervical strength in different directions: (a) Forward flexion, (b) extension, and (c) lateral right and left flexion were assessed using the Kinvent K-force handheld dynamometer (K-Force Dynamometer Muscle Controller, Kinvent, Montpellier, France) with twin handles (Kinvent, Montpellier, France) (Figure 1). During the cervical strength measurements, we also used (a) a stable chair (with a backrest and without an armrest); (b) a hard pillow, which was placed on the back of the chair; and (c) two Velcro straps for the stabilization of the participant during the test.

**Figure 2:**
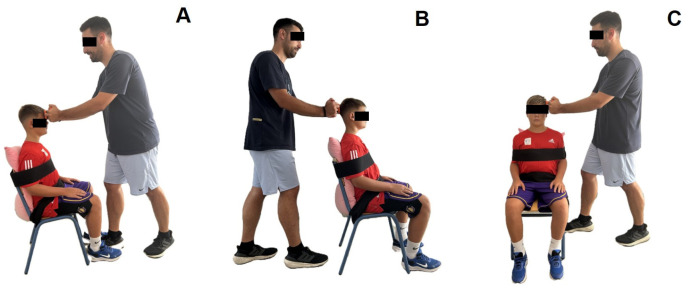
Position of the participant and the investigator during the measurement of forward flexion (**A**), extension (**B**), and right and left lateral flexion (**C**).

During the cervical strength measurements, particular attention was paid to the stable position of the participant and investigator. In more detail, the participant was placed in a sitting position with the back straight against the back of the chair, the hands relaxed on the thighs of the legs, and the feet flat on the floor (Figure 2). The investigator was placed in a standing position with one leg forward and arms with elbows slightly bent and locked (Figure 2). From this position, the investigator held the handheld dynamometer with both hands and placed it in the appropriate position for each tested movement direction. Specifically, the investigator placed the handheld dynamometer (a) on the center of the forehead, just superior to the eyebrows (for forward flexion direction), (b) slightly superior to the external occipital protuberance, and (c) on the lateral aspect of the head just superior to the ear (for lateral flexion direction). Then, he held resistance to the participants’ maximal effort [45].

The participant performed three maximal isometric contractions (duration of contraction: 5 s/repetition) in each direction of movement (1 min rest between each repetition and each testing direction), and the best score (in kg) was considered for analysis. During the measurements, we considered valid attempts those attempts that were carried out with a consistent application of force for five seconds. Attempts with no consistent application of force from the participants were not taken into account. Furthermore, the cervical flexion-to-extension ratio was calculated using the following equation:

cervical_F/E_ ratio = (cervical forward flexion strength/cervical extension strength) × 100.

### 2.4. Statistics

In our study, all statistical analyses were performed using the IBM SPSS Statistics v.28 software (IBM Corporation, Armonk, NY, USA). Initially, the intraclass correlation coefficient (ICC) was used to examine the test–retest reliability, where an ICC value less than 0.5 denotes poor reliability, an ICC value between 0.5 and 0.75 denotes moderate reliability, an ICC value between 0.75 and 0.90 denotes good reliability, and an ICC value greater than 0.90 denotes high reliability. The standard error of measurements (SEM) and the 95% limits of agreement (LOA) were also calculated using the following equations: SEM = SD × (1-ICC) and LOA = inter-trial mean difference ± 1.96 SD of the inter-trial difference [46]. The SEM was divided by the mean of two measurements and multiplied by 100 to provide a percentage value (SEM%) [42], where a SEM value less than 5% denotes high reliability, a value between 5% and 10% denotes good reliability, a value equal to 10% indicates moderate reliability, and above 10% denotes low reliability. The inter-trial agreement was also examined graphically by plotting the differences between the test and retest against their mean, according to the Bland and Altman approach [47]. The presence of heteroscedasticity was tested using the Pearson correlation test to examine whether the absolute inter-trial difference was associated with the magnitude of the measurement. Paired *t*-tests were also used to determine possible significant differences between the test and retest in all the cervical movement directions. Finally, independent *t*-tests were used to examine possible differences in cervical strength measurement between prepubertal and pubertal boys. The level of significance was set at *p* < 0.05.

## 3. Results

### 3.1. Prepubertal Boys

#### 3.1.1. Cervical Strength (Forward Flexion, Extension, and Lateral Flexion) in Prepubertal Boys

Paired *t*-tests revealed non-significant differences between test and retest values for forward flexion (*t*_19_ = 1.467; *p* = 0.159), extension (*t*_19_ = 1.738; *p* = 0.098), and left (*t*_19_ = 1.845; *p* = 0.087) and right (*t*_19_ = 0.300; *p* = 0.767) lateral flexion. The systematic bias was −0.34 for forward flexion, −0.51 for extension, −0.06 for right lateral flexion, and −0.36 for left lateral flexion. Furthermore, the ICC (0.81–0.90) and SEM% (5.82–8.62) values denoted good reliability for all movement directions. Test and retest values (mean ± SD) and absolute and relative reliability indices (ICC, SEM, SEM%, and 95% LOA) for prepubertal boys are presented in Table 2.

**Figure 3:**
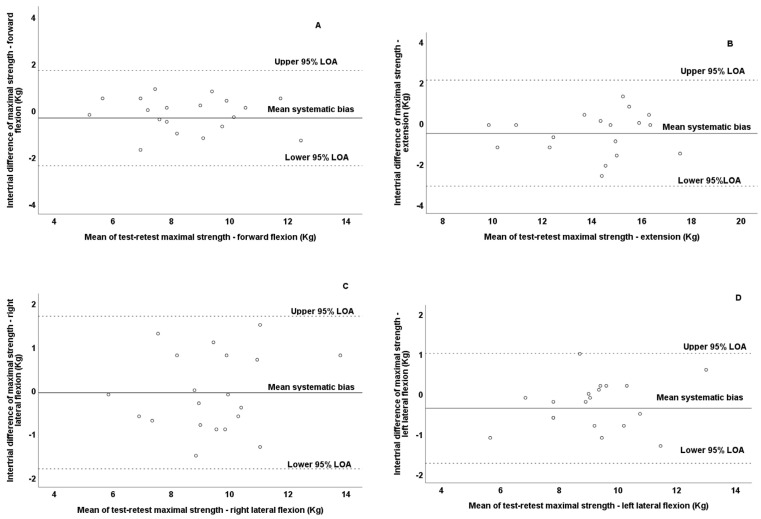
Bland–Altman plots of maximal isometric cervical strength during forward flexion (**A**), extension (**B**), right lateral flexion (**C**), and left lateral flexion (**D**) in test and retest measurements of prepubertal boys. The central solid line characterizes the mean difference between the test and retest values (systematic bias), while the upper and lower dashed lines characterize the upper and lower 95% limits of agreement—LOA.

Additionally, it should be mentioned that no presence of heteroscedasticity was observed since the absolute intertrial difference (systematic bias) was not associated with the magnitude of the measurement according to Pearson correlation test for forward flexion (*r* = −0.281; *p* = 0.230), extension (*r* = 0.114; *p* = 0.633), and right (*r* = 0.169; *p* = 0.476) and left (*r* = 0.002; *p* = 0.993) lateral flexion. Thus, all variables were found to be homoscedastic. Bland–Altman plots graphically present the reliability patterns for the assessment of cervical strength in all movement directions (Figure 3). According to the Bland–Altman plots, the differences between the test–retest values for all observations were within the defined 95% LOA of all tested variables. However, it should be noted that the observations in forward flexion demonstrated the least dispersion in the Bland–Altman plots (Figure 3A), while the lateral flexion demonstrated the greatest dispersion (Figure 3C,D).

#### 3.1.2. Cervical Flexion-to-Extension Ratio in Prepubertal Boys

Paired *t*-tests revealed non-significant differences between the test (61.07 ± 11.49%) and retest (61.01 ± 9.28%) values of the flexion-to-extension ratio (*t*_19_ = 0.030; *p* = 0.976). The systematic bias was −0.05 for the flexion-to-extension ratio, while the ICC (0.71) and SEM% (9.11) values denoted moderate reliability.

**Figure 4:**
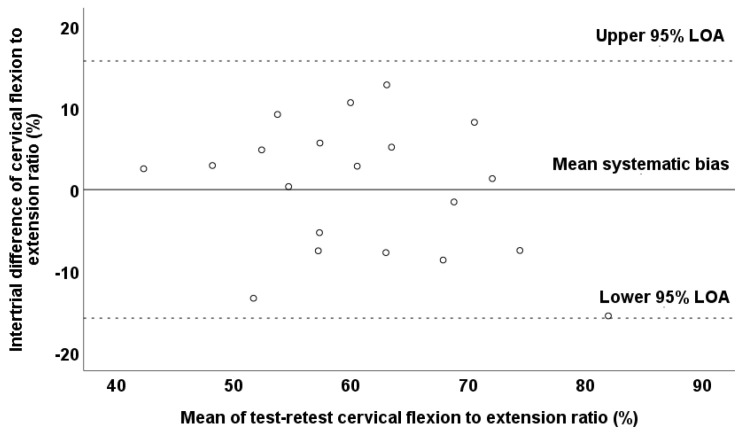
Bland–Altman plot of the cervical flexion-to-extension ratio values in test and retest measurements of prepubertal boys. The central solid line characterizes the mean difference between the test and retest values (systematic bias), while the upper and lower dashed lines characterize the upper and lower 95% limits of agreement—LOA.

Additionally, it should be mentioned that no presence of heteroscedasticity was observed since the absolute intertrial difference (systematic bias) was not associated with the magnitude of the measurement according to the Pearson correlation test (*r* = −0.296; *p* = 0.206), and thus, the variable was found to be homoscedastic. A Bland–Altman plot graphically presents the reliability pattern for the assessment of the cervical flexion–to–extension ratio (Figure 4). According to the Bland–Altman plot, the differences between the test–retest values for all observations were within the defined 95% LOA in the cervical flexion-to-extension ratio. However, it should be noted that the observations in the cervical flexion-to-extension ratio demonstrated great dispersion in the Bland–Altman plot (Figure 4).

### 3.2. Pubertal Boys

#### 3.2.1. Cervical Strength (Forward Flexion, Extension, and Lateral Flexion) in Pubertal Boys

Paired *t*-tests revealed non-significant differences between test and retest values for forward flexion (*t*_19_ = −1.086; *p* = 0.291), extension (*t*_19_ = 0.740; *p* = 0.468), and right (*t*_19_ = 1.935; *p* = 0.076) and left (*t*_19_ = −1.42; *p* = 0.173) lateral flexion. The systematic bias was 0.16 for forward flexion, −0.21 for extension, 0.60 for right lateral flexion, and 0.32 for left lateral flexion. Furthermore, the ICC (0.90–0.96) and SEM% (3.79–5.49) values denoted high reliability for all movement directions. Test and retest values (mean ± SD) and absolute and relative reliability indices (ICC, SEM, SEM%, and 95% LOA) for pubertal boys are presented in Table 3.

**Figure 5:**
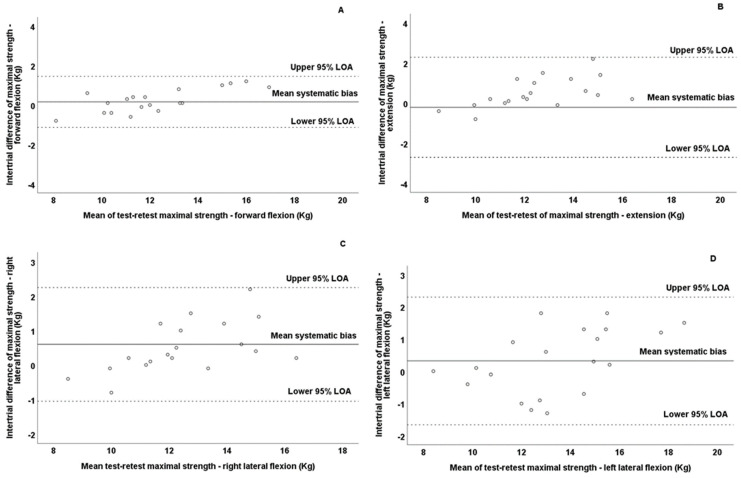
Bland–Altman plots of the maximal isometric cervical strength during forward flexion (**A**), extension (**B**), right lateral flexion (**C**), and left lateral flexion (**D**) in test and retest measurements of pubertal boys. The central solid line characterizes the mean difference between the test and retest values (systematic bias), while the upper and lower dashed lines characterize the upper and lower 95% limits of agreement—LOA.

Additionally, it should be mentioned that no presence of heteroscedasticity was observed since the absolute intertrial difference (systematic bias) was not associated with the magnitude of the measurement, according to Pearson correlation tests, for forward flexion (*r* = 0.110; *p* = 0.650), extension (*r* = −0.118; *p* = 0.621), and right (*r* = 0.234; *p* = 0.094) and left (*r* = 0.201; *p* = 0.085) lateral flexion. Thus, all variables were found to be homoscedastic. Bland–Altman plots graphically present the reliability patterns for the assessment of cervical strength in all movement directions (Figure 5). According to the Bland–Altman plots, the differences between the test–retest values for all observations were within the defined 95% LOA of all tested variables. However, it should be noted that the observations in forward flexion demonstrated the least dispersion in the Bland–Altman plots (Figure 5A), while the lateral flexion demonstrated the greatest dispersion (Figure 5C,D).

#### 3.2.2. Cervical Flexion-to-Extension Ratio in Pubertal Boys

Paired *t*-test revealed a non-significant difference between the test (66.01 ± 8.75%) and retest (67.56 ± 11.3%) values for the flexion-to-extension ratio (*t*_19_ = −1.04; *p* = 0.313). The systematic bias was 1.55 for the flexion-to-extension ratio, while the ICC (0.78) and SEM% (7.03) values denoted moderate to good reliability.

**Figure 6:**
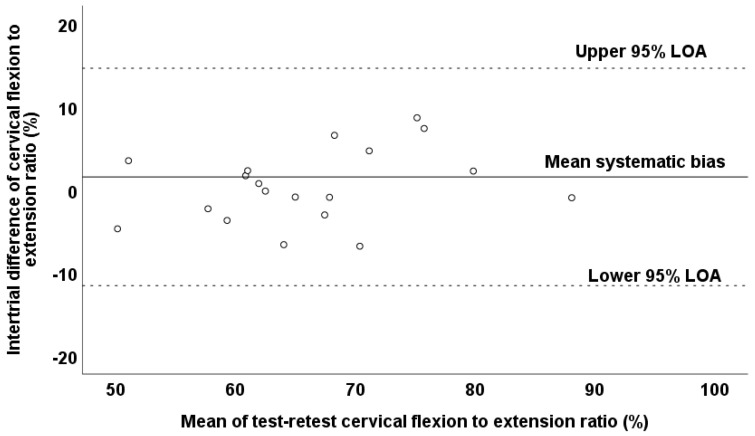
Bland–Altman plot of the cervical flexion-to-extension ratio values in the test and retest measurements. The central solid line characterizes the mean difference between the test and retest values (systematic bias), while the upper and lower dashed lines characterize the upper and lower 95% limits of agreement—LOA.

Additionally, it should be mentioned that no presence of heteroscedasticity was observed since the absolute intertrial difference (systematic bias) was not associated with the magnitude of the measurement according to the Pearson correlation test (*r* = 0.394; *p* = 0.086), and thus, the variable was found to be homoscedastic. A Bland–Altman plot graphically presents the reliability pattern for the assessment of the cervical flexion-to-extension ratio (Figure 6). According to the Bland–Altman plot, the differences between the test–retest values for all observations were within the defined 95% LOA of the cervical flexion-to-extension ratio. However, it should be noted that the observations of the cervical flexion-to-extension ratio demonstrated great dispersion in the Bland–Altman plot (Figure 6).

### 3.3. Comparison Between Prepubertal and Pubertal Boys

According to the reliability results, prepubertal boys reported lower relative and absolute reliability indices (ICC = 0.81–0.90; SEM% = 5.82–8.62) compared to pubertal boys (ICC = 0.90–0.96; SEM% = 3.79–5.49) in cervical strength measurement (forward flexion, extension, and lateral flexion).

Furthermore, independent *t*-tests showed significant differences in cervical strength values between prepubertal and pubertal boys for both testing occasions (test and retest). In more detail, *t*-tests showed significant differences in maximal isometric strength for forward flexion (*t*_38_ = −3.41; *p* = 0.000 in test/*t*_38_ = −5.57; *p* = 0.000 in retest), extension (*t*_38_ = −4.25; *p* = 0.000 in test/*t*_38_ = −5.34; *p* = 0.000 in retest), and right and left lateral flexion (*t*_38_ = −3.01 to −3.83; *p* = 0.000 in test/*t*_38_ = −5.23 to −5.99; *p* = 0.000 in retest) between prepubertal and pubertal boys, where pubertal boys demonstrated greater cervical strength values than prepubertal boys. However, in cervical flexion-to-extension ratio values, no significant differences were found between prepubertal and pubertal boys (*t*_38_ = −4.94 to −2.00; *p* = 0.135–0.07).

### 3.4. Total Sample

#### 3.4.1. Cervical Strength (Forward Flexion, Extension, and Lateral Flexion) in the Total Sample

Paired *t*-tests revealed non-significant differences between test and retest values for forward flexion (*t*_39_ = 0.637; *p* = 0.529), extension (*t*_39_ = 1.77; *p* = 0.084), and right (*t*_39_ = −1.86; *p* = 0.07) and left (*t*_39_ = 0.12; *p* = 0.901) lateral flexion. The systematic bias was −0.09 for forward flexion, −0.36 for extension, 0.27 for right lateral flexion, and −0.02 for left lateral flexion. Furthermore, the ICC (0.93–0.96) and SEM% (5.37–6.05) values denoted good/high reliability for all movement directions. Test and retest values (mean ± SD) and absolute and relative reliability indices (ICC, SEM, SEM%, and 95% LOA) for the total sample are presented in Table 4.

Additionally, it should be mentioned that no presence of heteroscedasticity was observed since the absolute intertrial difference (systematic bias) was not associated with the magnitude of the measurement, according to Pearson correlation tests, for forward flexion (*r* = 0.233; *p* = 0.148), extension (*r* = 0.06; *p* = 0.709), and right (*r* = 0.214; *p* = 0.095) and left (*r* = 0.211; *p* = 0.075) lateral flexion. Thus, all variables were found to be homoscedastic.

#### 3.4.2. Cervical Flexion-to-Extension Ratio in the Total Sample

Paired *t*-test revealed a non-significant difference between test (63.54 ± 10.38%) and retest (64.29 ± 10.71%) values for the flexion-to-extension ratio (*t*_39_ = −0.643; *p* = 0.524). The systematic bias was 0.75 for the flexion-to-extension ratio, while the ICC (0.76) and SEM% (8.08) values denoted moderate reliability.

Additionally, it should be mentioned that no presence of heteroscedasticity was observed since the absolute intertrial difference (systematic bias) was not associated with the magnitude of the measurement according to the Pearson correlation test (*r* = 0.047; *p* = 0.775), and thus, the variable was found to be homoscedastic.

## 4. Discussion

This study examined the test–retest reliability of maximal isometric cervical strength (in different movement directions: extension, forward flexion, and right–left lateral flexion), separately in prepubertal and pubertal untrained boys (comparing the two age groups) and pooled in the total sample, by using various indices of relative (ICC) and absolute reliability (SEM, SEM%, and 95% LOA). The results showed that the isometric strength of cervical muscles, using the Kinvent handheld dynamometer, can be reliably assessed in both prepubertal and pubertal untrained boys. It should be, however, mentioned that prepubertal boys displayed slightly lower relative and absolute reliability compared to pubertal boys. Additionally, the assessment of the cervical flexion-to-extension ratio demonstrated moderate reliability in prepubertal and pubertal boys, and thus, it should be interpreted with caution.

To the best of our knowledge, during the developmental years, only one study [40] has examined the reliability of cervical strength measurement using a handheld dynamometer. The results of that study [40] are generally in line with those of the present study, indicating that cervical strength can be measured reliably using a handheld dynamometer in the developmental years. It should be mentioned, however, that the ICC values of Stępień et al. [40] were higher (ICC = 0.98–0.99) than those observed in the present study (ICC = 0.81–0.90 in prepubertal boys and ICC = 0.90–0.96 in pubertal boys, depending on the testing direction). The discrepancies in the ICC values between the studies (the present study and Stępień et al.’s study) may be due to different factors that could affect the reliability results, such as the subject’s characteristics, as well as the testing protocol, position, and device. Regarding the subjects’ characteristics, in the present study, we used and compared separate groups of prepubertal and pubertal untrained boys, while in the study by Stępień et al. [40], the reliability results are presented in a mixed sample of healthy and sick (spinal muscular atrophy of different severities) children and adolescents (5–16 years old, including boys and girls). In the present study, prepubertal boys reported lower relative and absolute reliability indices (ICC = 0.81–0.90; SEM% = 5.82–8.62) compared to pubertal boys (ICC = 0.90–0.96; SEM% = 3.79–5.49) in cervical strength measurement, enhancing the importance of proper familiarization with the cervical strength measurement before primary testing, especially in children. However, when we examined the reliability of the total sample, our findings (ICC = 0.93–0.96) were similar to those of Stępień et al. [40]. Although other studies on cervical strength measurement have not analyzed reliability separately for age groups during the developmental years (children vs. adolescents), in comparison with our findings, previous studies with different strength measurements (i.e., isometric handgrip strength) have also shown the significant effect of age on reliability [41,42]. Indeed, Svenson et al. [42] reported that handgrip strength measurement (using the best of three trials) was more reliable in a 14-year-old group (ICC = 0.96, SEM%: 5.2) than in a 10-year-old group (ICC = 0.78, SEM%: 12.5). Additionally, Molenaar et al. [41] demonstrated that the smallest detectable difference expressed as a percentage of the maximum voluntary contraction decreased with increasing age, indicating higher reliability with increasing age (from 4 to 12 years old) in handgrip strength measurement. The aforementioned studies [41,42] mentioned that differences in mood, motivation, and attention between testing occasions, learning effects, and the maturity of the nervous system, as well as biomechanical factors, may account for these differences in the reliability of strength measurement among age groups. The possible differences in strength levels could also influence the reliability of the measurement. In the present study, we found significant differences in cervical strength measurements during forward flexion, extension, and lateral flexion between prepubertal and pubertal boys, where pubertal boys showed greater strength levels in all movement directions.

Another important differentiation between our study and that of Stępień et al. [40], which may explain the different reliability values in cervical strength measurement, is the testing position (the sitting position in the present study vs. the side-lying position in Stępień et al.’s study). In the scientific literature, during the developmental years, no previous study has examined the effect of testing position on the reliability of cervical strength measurement using a handheld dynamometer; however, a previous study on adults showed that the testing position affects the reliability of measurement [33]. In more detail, Krause et al. [33], with healthy adults (21–30 years old), examined two different positions (the lying and sitting positions) and showed significant differences in cervical strength values, as well as in the reliability of the measurement. Regarding the reliability of the cervical strength measurement, the lying position showed greater ICC values (0.89–0.95) compared to the sitting position (0.63–0.90) [33]. The authors of the above study [33] hypothesized that the use of a lying position during cervical strength measurement, compared to the sitting position, provides the examiner a mechanical advantage because the examiner can assume a position using body weight to effectively provide resistance against the force of the participant.

Despite the advantages of the lying position according to some researchers, several studies in the scientific literature have selected the sitting position during the measurement of cervical strength using different testing devices (i.e., fixed dynamometers, isokinetic dynamometers, and handheld dynamometers) [24,25,32,33,34,45,48,49]. A previous study [48] mentioned that the selection of a sitting position during cervical strength measurement is more comfortable for the examinees and easily ensures the neutral positioning of the head during cervical strength measurement. At this point, it should be mentioned that the stabilization of the participants during cervical strength measurement using a handheld dynamometer from a sitting position is of crucial importance. The stabilization of the participants’ bodies differs between previous studies in the scientific literature. For example, in one study, the participants’ bodies were stabilized by the examiner’s hand [34], while other testing protocols used a table that was positioned against a chair [33,49]. In the present study, we placed great emphasis on stabilization, and we used two Velcro straps to keep the participants’ body trunks steady to avoid the associative work of the trunk muscles during the measurement process that could affect the reliability of the measurement. The reliability results of the present study are in accordance and comparable with the results of previous studies (using fixed load cell strain gauges or force transducers) [24,25] on the developmental years that selected the sitting position for the evaluation of cervical strength measurement and reported good to high reliability [0.86–0.93].

An additional factor that could affect the results of studies is the calculated reliability indicators. In our study, in addition to the presentation of the relevant reliability indicators (ICC), absolute reliability indicators (SEM, SEM%, and 95% LOA) were also used. It is important to mention that relative reliability indicators (e.g., ICC) are more “sensitive” and are influenced by the range of measured values [46]. For this reason, the international literature proposes the use of absolute reliability indicators (e.g., SEM, SEM%, and 95% LOA), which are not affected by the range of measured values [46]. Therefore, the combined use and presentation of relative and absolute reliability indicators offers a more correct and comprehensive picture of the testing protocol’s reliability [46]. The only previous study [40] that examined the reliability of cervical strength measurement using a handheld dynamometer in developmental years presented only ICC reliability values, without any indicator of absolute reliability to compare with our results. On the other hand, the few other studies [23,24,25] that have examined the reliability of cervical strength in the developmental years, using fixed devices, have presented indices of relative and absolute reliability.

In our study, except for the measurement of maximal cervical strength, one more index that was calculated was the cervical flexion-to-extension ratio. The evaluation and calculation of this parameter may provide a more complete picture of the proper function, stability, and balance of the cervical spine. There is a notion that the muscular imbalance between the strength of the cervical extensor and flexor muscles can be negatively correlated with the stabilization of the cervical spine [50,51]. To the best of our knowledge, the reliability of the cervical flexion-to-extension ratio has not been previously assessed in the developmental years, so direct comparison with other studies is not possible. It should be noted that the reliability scores that we observed for the assessment of the cervical flexion-to-extension ratio were lower (ICC = 0.71 in prepubertal boys and 0.78 in pubertal boys) compared to those for cervical strength (ICC = 0.81–0.90 in prepubertal boys and ICC = 0.90–0.96 in pubertal boys)). Our results support earlier findings in adults that examined the reliability of the cervical flexion-to-extension ratio and reported lower reliability compared to cervical strength values [35,49]. The lower reliability values of the flexion-to-extension ratio can be explained by the fact that it is a composite of two absolute values, each possibly varying in the same or a different direction with reassessment, resulting in error propagation. Thus, it seems that the assessment of the cervical flexion-to-extension ratio should be interpreted with more caution, independent of age.

The present study has some limitations that could affect its generalization. First of all, the results of the present study are limited to healthy boys (children and adolescents). Future studies could be important in examining the reliability of cervical strength measurement (using a handheld dynamometer) in other pediatric populations (i.e., girls, athletic population, children with cervical pain, or athletes at risk of injury). Furthermore, an important limitation of this study is the sample size. Before the start of the study, we estimated, using the formula of Walter et al. [52], that a sample size of 20 participants per age group would be adequate for this study (power: 80%; minimum acceptable reliability—ICC: 0.7; expected reliability—ICC: 0.90). However, after the completion of the study, in some cases, we did not reach the expected ICC value. For this reason, future studies with a greater sample size (than that of the present study) per age group could reinforce the findings of the present study. Additionally, the results of the present study are limited to the testing position used (sitting position); future studies could examine and compare the reliability of cervical strength measurement using different testing positions in a pediatric population. Moreover, in the present study, we examined the test–retest reliability of cervical strength measurement in prepubertal and pubertal boys using one investigator, without examining the inter-rater reliability. To the best of our knowledge, during the developmental years, no previous study has examined the inter-rater reliability of cervical strength measurement using a handheld dynamometer. Future studies could examine the inter-rater reliability of cervical strength measurement in a pediatric population. Finally, future studies could examine different anatomical factors (e.g., the length of the cervical spine) or psychological confounders (e.g., motivation and attention span) that could affect strength performance during the developmental years.

## 5. Conclusions

In conclusion, maximal isometric cervical strength can be measured reliably from the sitting position using the Kinvent handheld dynamometer on prepubertal and pubertal untrained boys. These reliable testing protocols for cervical strength measurement may be used by coaches, physical conditioning trainers, and healthcare professionals to design, implement, and guide specific training programs for the cervical joint. In the present study, we observed slightly lower reliability in prepubertal compared to pubertal boys. The assessment of the cervical flexion-to-extension strength ratio demonstrated moderate reliability, emphasizing the need for a more careful interpretation of this parameter in the developmental years.

## Figures and Tables

**Table 1:** Age and anthropometric characteristics of the sample (mean ± standard deviation).

Variables	Prepubertal (*n* = 20)	Pubertal (*n* = 20)
Age (yrs)	9.10 ± 0.61	13.60 ± 0.60
Body height (m)	1.38 ± 0.08	1.65 ± 0.10
Body mass (Kg)	37.04 ± 10.10	61.10 ± 14.30
Biological age (Tanner Stage *)	I	III–IV

* Tanner stage is coded as I, II, III, IV, or V.

**Table 2:** Test–retest reliability indices of maximal isometric cervical strength in prepubertal boys.

	Test (kg)	Retest (kg)	ICC	SEM (kg)	SEM%	95% LOA	
						Lower	Upper
Forward flexion	8.88 ± 2.16	8.54 ± 1.88	0.86	0.75	8.62	−2.38	1.70
Extension	14.52 ± 2.13	14 ± 2.27	0.81	0.97	6.78	−3.08	2.06
Right lateral flexion	9.42 ± 1.74	9.36 ± 1.89	0.88	0.62	6.58	−1.80	1.68
Left lateral flexion	9.45 ± 1.70	9.10 ± 1.70	0.90	0.54	5.82	−1.73	1.02

ICC: intraclass correlation coefficient; 95% LOA: 95% limits of agreement; SEM: standard error of measurement; SEM%: standard error of measurement expressed as a percentage value.

**Table 3:** Test–retest reliability indices of maximal isometric cervical strength in pubertal boys.

	Test (kg)	Retest (kg)	ICC	SEM (kg)	SEM%	95% LOA	
						Lower	Upper
Forward flexion	12.29 ± 2.17	12.45 ± 2.52	0.96	0.47	3.79	−1.13	1.45
Extension	18.76 ± 3.21	18.55 ± 3.06	0.92	0.89	4.75	−2.70	2.28
Right lateral flexion	12.42 ± 1.96	13.02 ± 2.50	0.90	0.70	5.49	−1.05	2.25
Left lateral flexion	13.28 ± 2.41	13.60 ± 2.90	0.93	0.73	5.41	−1.66	2.30

ICC: intraclass correlation coefficient; 95% LOA: 95% limits of agreement; SEM: standard error of measurement; SEM%: standard error of measurement expressed as a percentage value.

**Table 4:** Test–retest reliability indices of maximal isometric cervical strength in the total sample.

	Test (kg)	Retest (kg)	ICC	SEM (kg)	SEM%	95% LOA	
						Lower	Upper
Forward flexion	10.58 ± 2.75	10.49 ± 2.95	0.95	0.64	6.05	−1.83	1.65
Extension	16.64 ± 3.44	16.28 ± 3.52	0.93	0.92	5.59	−2.87	2.15
Right lateral flexion	10.92 ± 2.38	11.19 ± 2.87	0.94	0.64	5.82	−1.53	2.07
Left lateral flexion	11.37 ± 2.83	11.35 ± 3.27	0.96	0.61	5.37	−1.84	1.80

ICC: intraclass correlation coefficient; 95% LOA: 95% limits of agreement; SEM: standard error of measurement; SEM%: standard error of measurement expressed as a percentage value.

## Data Availability

The data are unavailable due to privacy or ethical restrictions.
